# Educating EVs to Improve Bone Regeneration: Getting Closer to the Clinic

**DOI:** 10.3390/ijms23031865

**Published:** 2022-02-07

**Authors:** Arantza Infante, Natividad Alcorta-Sevillano, Iratxe Macías, Clara I. Rodríguez

**Affiliations:** 1 Stem Cells and Cell Therapy Laboratory, Biocruces Bizkaia Health Research Institute, Cruces University Hospital, Plaza de Cruces S/N, 48903 Barakaldo, Spain; arantza.infantemartinez@osakidetza.eus (A.I.); natividad.alcortasevillano@osakidetza.eus (N.A.-S.); iratxe.maciasgarcia@osakidetza.eus (I.M.); 2 Department of Cell Biology and Histology, University of Basque Country (UPV/EHU), 48940 Leioa, Spain

**Keywords:** extracellular vesicles, osteoanabolic, bone regeneration, MSCs, miRNAs

## Abstract

The incidence of bone-related disorders is continuously growing as the aging of the population in developing countries continues to increase. Although therapeutic interventions for bone regeneration exist, their effectiveness is questioned, especially under certain circumstances, such as critical size defects. This gap of curative options has led to the search for new and more effective therapeutic approaches for bone regeneration; among them, the possibility of using extracellular vesicles (EVs) is gaining ground. EVs are secreted, biocompatible, nano-sized vesicles that play a pivotal role as messengers between donor and target cells, mediated by their specific cargo. Evidence shows that bone-relevant cells secrete osteoanabolic EVs, whose functionality can be further improved by several strategies. This, together with the low immunogenicity of EVs and their storage advantages, make them attractive candidates for clinical prospects in bone regeneration. However, before EVs reach clinical translation, a number of concerns should be addressed. Unraveling the EVs’ mode of action in bone regeneration is one of them; the molecular mediators driving their osteoanabolic effects in acceptor cells are now beginning to be uncovered. Increasing the functional and bone targeting abilities of EVs are also matters of intense research. Here, we summarize the cell sources offering osteoanabolic EVs, and the current knowledge about the molecular cargos that mediate bone regeneration. Moreover, we discuss strategies under development to improve the osteoanabolic and bone-targeting potential of EVs.

## 1. Introduction

Bone is a dynamic organ that is constantly remodeling to ensure a constant net bone mass within an organism. This is achieved by two opposite and balanced phases: bone formation and bone resorption, carried out by osteoblasts and osteoclasts, respectively. Moreover, bone has a self-repairing ability, and therefore when an injury occurs, the damaged part regains its original structure and mechanical strength by the activation of the bone healing process. However, under certain situations, such as critical-sized bone defects (defined as those that will not heal spontaneously during the life of a patient), bone loss due to bone-related diseases, lack of vascularization, infections, and tumors, this ability is not enough and requires clinical intervention [1]. In the case of osteoporosis (OP), a highly prevalent metabolic disease affecting more than 200 million of patients worldwide, each year more than 8.9 million fractures are reported globally [2]. In the EU, the cost of OP in 2019, including pharmacological intervention, entailed more than €56 billion, doubling that needed in 2010 [3,4].

Bone tissue is highly demanded in clinics, and after blood, is the second most transplanted tissue worldwide [5]. Thus, currently, the most successful intervention to treat bone defects is still bone grafting, a strategy first outlined in the early 1900s [6]. Bone autografts are the gold standard in bone regeneration procedures since they avoid rejection from the patient’s immune system. Unfortunately, autologous bone supply is limited and the need of additional surgery for bone extraction increases the risk of infections and morbidity. Another option is the use of allografts (from a human donor) or xenografts (from large animals like pigs or bovines), which nevertheless entail some risk of pathogen transmission; more importantly, these bone implants have shown poor bone regeneration abilities [7]. All in all, there is an urgent need to discover new, effective therapies to boost bone regeneration to satisfy the growing world population (progressively more aged) affected by bone conditions. 

On this basis, the field of bone tissue engineering has emerged, focused on developing “bone substitutes” that mimic the bone tissue features, usually formed by a 3D scaffold and bone-relevant cell types, which are able to promote osteogenic differentiation in host tissue without any adverse inflammatory response [8]. The features of mesenchymal stem cells (MSCs), such as their capacity to undergo osteogenic differentiation, immunomodulatory potential, and trophic effects, make them quite attractive components for these bone constructs [8,9]. In fact, the use of MSCs-based therapies in clinics is gaining interest in the field of bone regeneration due to the clinical improvements exhibited by patients affected by bone-related diseases, such as Osteogenesis Imperfecta (OI) after MSCs administrations [8,10,11,12]. However, there are still some limitations to the clinical translation of MSCs, such as the large number of cells that are required as well as the high cellular heterogeneity, even within populations of MSCs from the same donor. In addition, other factors, such as cell culture conditions, cell source, and donor age, have determined the variable outcomes shown in different clinical trials using MSCs [13]. Regarding the mode of action of MSCs therapy in bone regeneration, quite revealing findings point to the paracrine mechanisms elicited by these cells, rather than the initially expected cell engraftment and subsequent osteogenic differentiation [10,14]. Moreover, a set of experiments performed in OI animal models, suggested that the extracellular vesicles (EVs) secreted by MSCs could be mediating the recovery of bone phenotypes observed in OI patients subjected to MSCs therapy [15]. 

EVs are small, lipid membrane delimited particles secreted by most cell types and present in several biological fluids, such as blood and urine, that play a key role in cell-to-cell communication [16]. This paracrine crosstalk is mediated by the EVs’ cargo, an array of bioactive molecules including proteins, lipids, and nucleic acids, that interestingly exhibit a parent-cell-specific signature [17,18]. Thus, through specific surface molecule interaction, EVs can be uptaken by target cells and modify their biology/fate [19]. Attending to their size, the EVs population can be divided into exosomes (diameters of 30–200 nm), microvesicles (diameters of 200–1000 nm), and apoptotic bodies (diameters > 1000 nm) [19,20]. The biogenesis of EVs is a complex process and currently it is accepted that each EV subtype can be originated by two predominant pathways: the endosomal pathway, through multivesicular endosome fusion, or by the outward budding and fission of the plasmatic membrane [21]. The fact that EVs carry functional molecules that can modulate target cell responses opens the possibility of using EVs as next-generation drug delivery platforms, a vision strongly supported by their low immunogenicity. Thus, EVs are known to escape from immune clearance when systemically administered, due to the expression of surface molecules, such as CD47, which mediates the “don’t eat me” signal that blocks phagocytosis [22]. Bone-relevant cell types have been shown to secrete EVs that regulate bone homeostasis, and in addition, recent evidences suggest that EV therapy is at least as efficient as cell therapy in eliciting bone regeneration in large bone defect animal models [19,23,24]. Thus, the use of EVs as osteoanabolic delivery systems could be a reliable clinical approach for bone regeneration [25]. 

In this review, we recapitulate the current knowledge about the most suitable cell sources to obtain osteoanabolic EVs along with the described mechanisms triggered by EVs in bone target cells. We also discuss the limitations of EVs when intended for bone regeneration and the emerging strategies that are currently under intensive research to counteract these drawbacks, and we obtain educated EVs with enhanced bone regenerative potential.

## 2. EV Sources for Bone Regeneration and Mechanisms of Action 

From a clinical perspective, most research in the field of bone regeneration and EVs, which has grown exponentially in the last years, has focused on either exosomes or microvesicles. However, these EV populations are not fully characterized, in spite of the remarkable efforts made by the International Society of Extracellular Vesicles (ISEV) to determine the minimal information for studies regarding EVs (Minimal information for studies of extracellular vesicles 2018 (MISEV2018): a position statement of the International Society for Extracellular Vesicles and update of the MISEV2014 guidelines, doi:10.1080/20013078.2018.1535750). Following these recommendations, in this review we will use EVs as a broad term encompassing all the vesicles secreted by cells [26].

**Figure 1:**
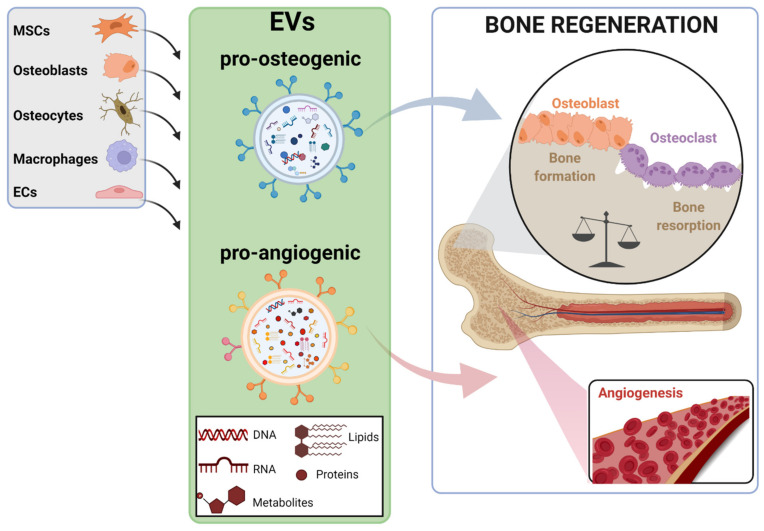
Illustration of the bone-relevant cell types known to secrete EVs that promote bone regeneration. Pro-osteogenic and pro-angiogenic EVs, determined by their specific cargo, are produced by cell types present in bone microenvironment, such as mesenchymal stem cells (MSCs), osteoblasts, osteocytes, macrophages, and endothelial cells (ECs). Pro-osteogenic EVs stimulate MSCs and osteoblasts differentiation, inducing the bone formation process, while pro-angiogenic EVs elicit the formation of new blood vessels in bone tissue. Both processes are essential to conduct a successful regeneration of bone tissue. The figure was created with BioRender.com (accessed on 1 December 2021).

Bone resident cells, including bone-cell types (MSCs, osteoblasts, osteoclasts, and osteocytes) as well as other cell populations present in the bone microenvironment, such as endothelial cells (ECs) and macrophages, secrete EVs that mediate the continuous bone remodeling process (Figure 1) [23]. Interestingly, the cargo contained in the EVs reflects the biological function of parent cells. Thus, osteoclast-secreted EVs can inhibit osteoblast activity and therefore suppress bone formation, whereas the EVs secreted by osteoblastic-lineage cells, such as MSCs and osteoblasts, enhance osteoblast differentiation in vitro and promote bone regeneration in vivo [27,28]. Moreover, osteoblast-derived EVs also can be uptaken by osteoclasts, but the consequences triggered in these cells are conflicting. Thus, the promotion of osteoclasts’ differentiation in vitro and in vivo has been described, but also the inhibition of osteoclastogenesis, and therefore of bone resorption [29,30,31]. Current evidence, mainly obtained from basic and preclinical experimentation, strongly suggest that the specific cargo of EVs determines the signaling triggered in recipient cells. In this line, miRNAs contained in EVs are known to be transferred to target cells as a mechanism of genetic exchange between cells, playing a key role in regulating bone homeostasis [32]. Actually, a specific, dysregulated miRNAs signature has been described in plasma/serum EVs of some pathological conditions, including bone disease. This is quite valuable in silent pathologies, such as OP, which has no clinical manifestation until a fracture occurs, being that these circulating miRNAs, proposed as potential biomarkers, are capable of predicting the risk of fracture [33,34].

Next, we will discuss the current knowledge about the bone-relevant cell types that secrete osteoanabolic EVs and the current identified mechanisms, mainly driven by miRNAs (and some proteins, to a lesser extent) (Table 1), by which these EVs exert their function in target cells.

### 2.1. EVs Derived from MSCs 

MSCs represent a promising cell population for clinical application in bone diseases, mainly due to the paracrine properties they exert [10,13]. However, the therapeutic potential of this advanced therapy may be limited by several factors. Thus, the heterogeneity of MSCs sources, isolation, and culture methods, in addition to the age of the MSCs’ donors, directly affect the defining features of MSCs, such as the proliferation, differentiation, and secretory abilities [47,48]. Interestingly, cell-free approaches based on the secreted products of cells (mainly MSCs), have also been proposed as therapeutic agents since they can achieve similar results to those elicited by cells themselves, overcoming the complexity that involves the administration to patients of a “live” treatment. In this context, the possibility of using EVs derived from MSCs (MSCs-EVs) as a therapy for bone regeneration is gaining interest in the scientific community [30,49].

MSCs present different capacities to induce bone regeneration depending on their original tissue, such as bone marrow (BM), adipose tissue (AT), and umbilical cord (UC) [50]. For instance, while BM-MSCs show better proliferation and differentiation potential in chondrocytes and osteoblasts, UC-MSCs appear to be more pro-angiogenic, transferring more blood supply during bone regeneration [51]. Therefore, it is not surprising that EVs secreted by MSCs isolated from different sources present different composition, characteristics, and even functional properties [52]. A recent comparative proteomic analysis revealed that EVs derived from BM-MSCs (BM-MSCs-EVs) were enriched in osteoanabolic proteins, whereas EVs from AT-MSCs (AT-MSCs-EVs) contained a high number of immune response proteins and EVs derived from UC-MSCs (UC-MSCs-EVs) were rich in proteins mediating endothelial homeostasis [53]. Moreover, the characterization of full small RNAome of MSC-EVs has demonstrated significant differences in the RNA composition (miRNAs and tRNAs) of EVs derived from AT-MSCs and BM-MSCs [54].

#### 2.1.1. EVs Derived from BM-MSCs

BM-derived MSCs show a great capacity to undergo osteogenic differentiation and therefore, as expected, BM-MSCs-EVs also exhibit potential to promote the proliferation of human osteoblasts and osteogenic differentiation of MSCs, both in vivo and in vitro [27]. Interestingly, there is evidence pointing to different efficacies of EVs depending on the anatomic origin of BM-MSCs, which in turn determines the osteogenic potential of MSCs. Thus, jawbone BM-MSCs exhibited superior osteogenic capacities to iliac crest BM-MSCs and as was foreseeable, jawbone BM-MSCs-EVs showed superior osteoanabolic abilities than iliac crest BM-MSCs-EVs [55].

Several miRNAs contained in BM-MSCs-EVs have been shown to play a key role in their osteoanabolic properties. That is the case of miR-335, which was enriched in BM-MSCs-EVs and mediated the bone healing process in mice bone fracture models. In recipient MSCs, miR-335 inhibited the in vitro expression of the pro-osteoclastogenic protein VAPB, promoting the activation of Wnt/β-catenin pathway, and therefore stimulating the osteogenesis of MSCs, plus, finally the in vivo fracture repair in mice models [35]. BM-MSCs-EVs also have been shown to promote fracture healing in mice mediated by enriched miR-25, which facilitates osteogenic differentiation, proliferation, and migration of osteoblasts by inhibiting RUNX2 degradation through downregulation of the ubiquitin ligase SMURF1[36].

Moreover, the miRNAs contained in BM-MSCs-EVs have also been suggested to mediate bone regeneration under pathological conditions. This is the case of a mice model of the rare bone disorder OI, that, after receiving BM-MSCs-EVs, showed an increase in bone growth [15]. Moreover, the authors showed that the removal of RNA molecules from the EVs, either by direct RNase treatment of EVs or by depleting the expression of miRNAs in MSCs, resulted in the secretion of EVs that failed to stimulate chondrocyte proliferation in vitro.

Interestingly, two specific conditions of BM-MSCs have been proposed to be of special relevance to determine the osteoanabolic abilities of their secreted EVs. The age of BM-MSCs is one of them: young BM-MSCs-EVs demonstrated enhanced in vitro and in vivo osteoanabolic properties when compared to EVs obtained from old counterparts. Thus, EVs isolated from aged rat BM-MSCs did not improve fracture healing as efficiently as EVs isolated from young BM-MSCs did. In vitro experiments showed a subset of upregulated miRNAs in EVs coming from aged BM-MSCs, hampering their inherent osteoanabolic potential. This way, miR-128-3p, which was upregulated in aged-EVs, inhibited SMAD5 in recipient MSCs, a known pro-osteogenic transcription factor [39]. The targeting of this miRNA by antagomiRs could therefore be an approach for bone fractures in aged patients.

The second specific condition determining the osteoanabolic potential of EVs is the osteogenic differentiation stage of BM-MSCs [56]. Strikingly, recent works have pointed out that EVs derived from MSCs in the late state of osteogenic differentiation (day 21) have increased osteoanabolic potential when compared to EVs from undifferentiated MSCs or MSCs undergoing early osteogenic differentiation (day 3) [57]. The different cargo composition of EVs depending on the differentiation stage of MSCs was a possible explanation for this finding. In fact, a specific pro-osteogenic miRNA, miR-101, exhibited a gradual expression modulation, rising throughout the process of osteogenic differentiation, in both MSCs and in their secreted EVs. miR-101 targeted FBXW7, an E3 ubiquitin ligase that represses osteogenic differentiation of MSCs. Other works have also reported these findings: EVs derived from MSCs from the late stages of osteogenic differentiation were enriched in a specific set of pro-osteogenic miRNAs, such as miR-10b, while the anti-osteogenic microRNAs miR-31, miR-144, and miR-221 were decreased [58,59,60,61,62].

The coupling of angiogenesis with osteogenesis that takes place in bone regeneration, initially described in 2014, has been highlighted as an essential mechanism to promote and sustain bone formation [63]. In fact, blood vessels bring oxygen and nutrients to the regenerating bone, and serve as a route for inflammatory cells, as well as cartilage and bone precursor cells, to reach the injury site [64]. Interestingly, BM-MSCs-EVs have been shown to promote angiogenesis in bone repair, thus accelerating the bone regeneration process. Thus, miR-29a contained in BM-MSCs-EVs was shown to be delivered into endothelial cells (ECs) in vitro, promoting their proliferation, migration, and tube formation abilities. Moreover, EVs isolated from BM-MSCs overexpressing miR-29a were shown to promote angiogenesis and osteogenesis in vivo, leading to an increased bone mass in mice [38]. VASH1, a negative regulator of angiogenesis, was identified as a direct target of miR-29a. Interestingly, the authors revealed that miR-29a was markedly decreased in EVs secreted by aged BM-MSCs when compared to EVs secreted by young counterparts. Observations implied that the downregulation of miR-29a in aged EVs could take part in the bone loss observed during aging [38].

Protein transfer between BM-MSCs-EVs and recipient cells, such as ECs, is another mechanism driving the activation of the bone regeneration elicited by EVs. That´s the case of Nidogenin1 (NID1), an extracellular matrix (ECM) scaffold protein mainly secreted by stromal cells. Thus, BM-MSCs-EVs were found to be enriched in NID1 but not the EVs secreted by other relevant cell types for bone regeneration, such as ECs. In vitro experiments showed that NID1, transferred via BM-MSCs-EVs to ECs, was bound to Myosin-10 in the cytoplasm of these cells, leading to the inhibition of the formation of focal adhesions. Consequently, these ECs showed increased migration and tube formation, a finding that was confirmed in mice models of femoral condylar defects. Thus, authors treated femoral defects with hydrogels composed of BM-MSCs-EVs with or without the expression of NID1, showing that the EVs not expressing NID1 were less efficient in promoting angiogenesis and bone repair [37].

#### 2.1.2. EVs Derived from UC-MSCs

Recent findings show that UC-MSCs-EVs promote bone regeneration by triggering different processes. One of them is the inhibition of the apoptosis that specific bone cells undergo under pathological conditions, such as disuse osteoporosis (DOP) and glucocorticoid-induced osteonecrosis of the femoral head (GIONFH). In the case of DOP, resulting from a lack or minimum mechanical loading, BM-MSCs isolated from hind limbs of DOP mice models showed less apoptosis after being treated in vitro with UC-MSCs-EVs. A concomitant bone mass restoration was also observed in these animal models, which was suggested as being a consequence of the reduced apoptosis of BM-MSCs after UC-MSCs-EVs treatment [40]. Moreover, after examining the miRNA expression in the hind limbs of mice receiving or not receiving UC-MSCs-EVs, the authors uncovered an upregulation of several miRNAs, among them miR-1263, exhibiting the highest upregulation. Further experiments, by silencing or mimicking the expression of miR-1263 in the UC-MSCs, and therefore in the secreted EVs, confirmed the role of miR-1263 in bone mass restoration in the DOP animal models. MiR-1263 was shown to target MOB1, an essential component of the Hippo signaling pathway, which is involved in the regulation of apoptosis and osteogenesis of MSCs.

Regarding GIONFH, miR-21 present in UC-MSCs-EVs was shown to repress dexamethasone-induced apoptosis in osteocytes in vitro and to mediate the prevention of GIONFH in animal models by targeting PTEN, a known inhibitor of the AKT signaling pathway, which in turn promotes cell survival [41]. MiR-365a-5p was also identified as enriched in UC-MSCs-EVs, and being able to induce osteogenesis and proliferation of osteoblasts in GIONFH animal models by targeting SAV1, another key component of the Hippo signaling pathway [42].

UC-MSCS-EVs have also demonstrated the ability to ameliorate bone loss in senile osteoporotic mice by promoting osteogenic differentiation of MSCs and inhibiting osteoclast formation in vitro. The bone anabolic effects of UC-MSCs-EVs were found to be mediated by miR-3960 [43].

The transfer of osteoanabolic proteins from UC-MSCs-EVs to target cells has also been identified as a mechanism promoting osteogenesis. This way, CLEC11A, a potent osteoanabolic secreted protein identified in 2016, was shown to be highly enriched in UC-MSCs-EVs when compared to the proteome of UC-MSCs [44,65]. CLEC11A contained in these EVs was suggested to be responsible for the amelioration of bone osteoporotic phenotypes in three mice models of OP, since in vitro studies indicated that the presence of CLEC11A in EVs was a determining factor for enhancing osteogenic differentiation of MSCs while inhibiting osteoclast’s activity [44].

#### 2.1.3. EVS Derived from AT-MSCs

The osteoanabolic potential of AT-MSCs-EVs has been shown in a number of studies. As observed in BM-MSCs, the differentiation state of AT-MSCs was also shown to affect the EVs’ cargo; those AT-MSCs undergoing osteogenic differentiation showed enriched miRNAs related with osteogenic differentiation [66]. However, when comparing the osteoanabolic potential of AT-MSCs-EVs with BM-MSCs-EVs, the obtained results are controversial. A recent study found that AT-MSCs-EVs showed the best performance for in vitro and in vivo chondrogenesis and osteogenesis when compared with BM-MSCs-EVs [67]. On the contrary, another recent study pointed to superior osteoanabolic potential of EVs derived from osteogenically induced BM-MSCs when compared to those obtained from osteogenically induced AT-MSCs [68].

### 2.2. EVs Derived from Osteocytes

Osteocytes, terminally differentiated osteoblasts embedded within the mineralized bone matrix, are known to coordinate bone remodeling by regulating both osteoblast and osteoclast functions mainly by paracrine mechanisms [69]. Interestingly, under specific mechanical cues, osteocytes have been shown to release a substantial amount of EVs containing proteins, such as sclerostin, RANKL and OPG, that enhance the MSCs’ recruitment to the bone damage site, thus promoting osteogenesis and bone regeneration [70,71].

### 2.3. EVs Derived from Endothelial Cells (ECs)

EVs from progenitor ECs, a heterogeneous cell population that resides in the BM and that differentiates into mature ECs upon vascular injury, have been shown to accelerate bone regeneration during distraction osteogenesis (DO), by stimulating angiogenesis in murine models of large bone defects [24]. MiR-126 contained in these EVs was suggested to be mediating DO due to the observation that in vitro miR-126 enhanced the proliferation, migration, and tube formation of ECs. As a mechanism, the authors showed that miR-126 targeted the expression of SPRED-1 in HUVECs, which in turn inhibits the pro-angiogenic Raf/ERK signaling pathway.

Strikingly, a recent study demonstrated that ECs-EVs seem to be more effective in targeting bone tissue than EVs derived from osteoblastic lineage cells, such as MSCs and osteoblasts [46]. These ECs-EVs were able to inhibit not only the osteoclast activity in vitro, but also the development of OP in an ovariectomized mice model (OVX mice) simulating post-menopausal OP. MiR-155, an enriched miRNA within EC-EVs, was identified to be responsible, at least in part, for inhibiting bone resorption in OVX mice by targeting key drivers of osteoclastogenesis, such as Spi1, Mitf, and Socs1 in BM-derived macrophages [46].

### 2.4. EVs Derived from Macrophages

Concerning immune cells, proper crosstalk between macrophages and MSCs is required during the whole process of bone healing, mainly driven by paracrine signaling [72]. Thus, within the first inflammatory stage, soon after the bone injury occurs, activated pro-inflammatory M1 macrophages release inflammatory and chemotactic factors that induce the recruitment of MSCs to the fracture site. Then, the transition of M1 macrophages to the anti-inflammatory M2 phenotype, supports the osteogenesis process of MSCs in the later stages of fracture healing [73]. Subsequently, this macrophage polarization also influences their EV cargo and related paracrine functions that mediate bone regeneration. In that way, EVs secreted by M1 polarized macrophages take part in the early phases of osteogenic differentiation and the EVs from the M2 phenotype foster continued bone regeneration [74]. The existence of polarization-specific miRNAs cargos in EVs from M1 and M2 macrophages has been suggested to be influencing their differential osteogenic signaling in MSCs [74].

## 3. Novel Strategies to Improve the Bone Regenerative Potential of EVs

The bone regenerative potential of EVs, especially if they come from MSCs and ECs, is an undeniable fact nowadays, supported (as we mentioned above) by a considerable number of basic and preclinical studies [75]. Nevertheless, the use of EVs as an advanced therapy for bone regeneration has been hampered mainly by two observations inherent to EV biology. First, the osteoanabolic potential of EVs is far from being optimum to achieve complete bone regeneration, and therefore these osteoanabolic abilities should be improved; second, osteoanabolic EVs do not mainly target bone tissue. Thus, upon intravenous administration in mice, EVs show a rapid (within the first hour) tissue distribution, accumulating mainly in the spleen, liver, lung, and kidneys [76,77]. Even so, there are also studies indicating the accumulation of MSCs-derived EVs in bone tissue, although to a lesser extent [78]. On the contrary, EVs coming from osteoclasts, known to negatively regulate bone formation by targeting and inhibiting osteoblasts, have shown acceptable intra-osseous accumulation in injected mice [28]. Therefore, current attempts pursuing the production of EVs with the maximum bone regenerative potential mainly rely on the enhancement of the EVs’ osteoanabolic abilities as well as on their bone cell targeting and, therefore, bone tissue.

### 3.1. Enhancing the Osteoanabolic Potential of EVs

MSCs undergoing osteogenic differentiation, especially those isolated from BM, have demonstrated acceptable bone regeneration properties due to two facts: their EVs show increased bone targeting potential and exhibit osteoanabolic-specific cargo [79,80,81]. Hence, it is not surprising that the vast variety of investigations focus on enhancing the osteogenesis of parent MSCs in order to achieve innate EVs with maximum osteoanabolic and bone targeting abilities.

#### 3.1.1. Preconditioning of Parent Cells

**Figure 2:**
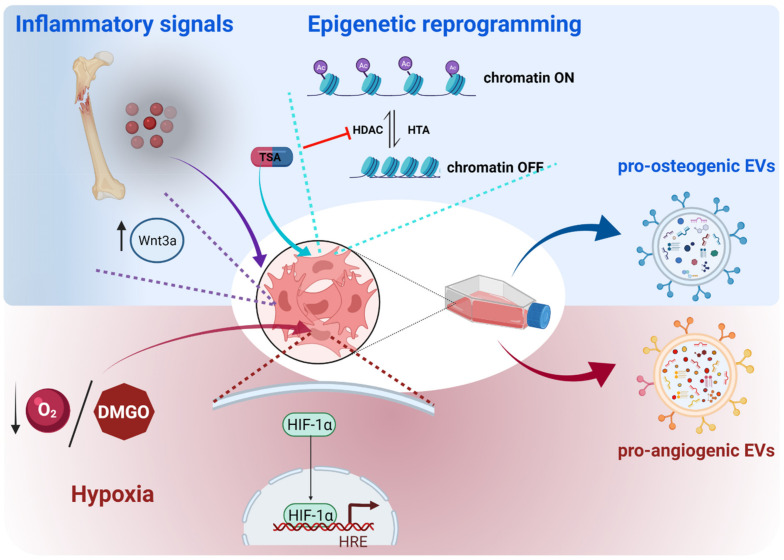
Preconditioning strategies to enhance the osteoanabolic potential of EVs. Pre-treatment of MSCs with inflammatory factors or histone deacetylase inhibitors enhance their osteogenic differentiation, whereas hypoxia conditions elicit pro-angiogenic responses in these cells, leading to the secretion of pro-osteogenic or pro-angiogenic EVs, respectively. Mechanistically, the mimicking of the bone inflammatory microenvironment after bone injury triggers the expression of the pro-osteogenic protein WNT3a in MSCs, which in turn, is enriched in the EVs secreted by these cells. The inhibition of histone deacetylases, such as via the use of thrichostatin A (TSA), elicits an epigenetic reprogramming of MSCs, ensuring an open conformation of chromatin and promoting the transcription of pro-osteogenic genes. The hypoxia simulation in MSCs, achieved by low oxygen cell culture or by chemical compounds (for instance dimethyloxaylglycine (DMOG)), induces the activation of the HIF-1α transcription factor, which drives the cell responses to hypoxia, among them being hypoxia-induced angiogenesis. The figure was created with BioRender.com (accessed on 1 December 2021).

Preconditioning of MSCs’ culture conditions, either by the addition of exogenous molecules (cytokines, growth factors, drugs) or by the optimization of physical factors (hypoxia or shear stress), has been proposed, as these strategies induce a robust osteogenic differentiation in MSCs in order to obtain highly osteoanabolic EVs (Figure 2) [82]. Along this line, the mimicking of the bone healing signaling milieu, such as that occurring in the inflammatory phase upon bone injury, has been demonstrated to be effective. Thus, when priming AT-MSCs with TNF-α, a specific pro-inflammatory molecule, the secreted EVs showed enhanced abilities in promoting the proliferation and osteogenic differentiation of human primary osteoblastic cells. Interestingly, an increase in WNT3a protein, a known inducer of osteogenesis, was detected in the cargo of these EVs [83].

Another strategy, the inhibition of deacetylation in MSCs, a process known as epigenetic reprogramming, is gaining attention in the field of bone regeneration [84]. Since deacetylation of histones by histone deacetylases (HDACs) induces a closed chromatin conformation and repression of transcription, inhibition of deacetylation by HDACs inhibitors (HDACis) favors the activation of transcription factors, among them the pro-osteogenic ones [85]. In this line, several studies point to the hyperacetylation of the chromatin through the use of HDACis to enhance the osteogenic potential of MSCs, both in vitro and in vivo [86]. Moreover, a quite recent study showed an enhancement of the therapeutic efficacy of MSCs-EVs for bone repair by treating parent MSCs with trichostatin A (TSA), an HDACi. In this context, TSA-treated MSCs and their secreted EVs showed enhanced osteogenic potential [87]. Interestingly, EVs isolated from MSCs differentiated to osteoblasts and treated with TSA exhibited some differential features, such as particle size and concentration. The cargo of EVs was also shown to be modified by TSA; proteins involved in transcriptional regulation were found to be upregulated when compared to EVs coming from untreated MSCs. In addition, an enhancement in RNA quantity, with an enrichment in pro-osteogenic microRNAs was also found. This differential cargo of TSA-MSCs-EVs was suggested to be the underlying mechanism mediating the enhanced osteoanabolic potential and bone repair that the TSA-MSCs-EVs exhibited.

On the other hand, upon in vitro culture conditions, MSCs are exposed to high O_2_ concentrations (21%), in contrast to the hypoxic milieu of the in vivo MSCs niche (2–8% O_2_). Under these high oxygen concentrations, MSCs loose important distinctive features, among them, their paracrine properties. Conversely, MSCs cultured under hypoxic conditions show increased production of EVs with an enhanced protein concentration when compared to normoxia-cultured MSCs [45,88]. Interestingly, two recent independent studies have shown that the EVs isolated from MSCs cultured under hypoxic conditions improve bone healing in preclinical models of bone defects [45,89]. Thus, GIONFH animal models showed improved bone regeneration after receiving EVs coming from hypoxia-MSCs compared to the ones coming from normoxia-MSCs. Strikingly, in vivo, the bone callus of the bone undergoing repair exhibited an increased neovascularization rather than increased osteogenesis. In vitro cultures showed that EVs from hypoxia-UC-MSCs were more easily uptaken by ECs, which in turn enhanced the expression of VEGF and showed improvements in proliferation, migration, and tube formation [45,89]. Furthermore, EVs from hypoxia-UC-MSCs showed a dysregulation of a number of miRNAs, the majority of them being upregulated. Among them, miR-126 (aforementioned to be also enriched in ECs-EVs; Table 1) was shown to be transferred to ECs, driving at least in part the enhanced proliferative, migratory, and angiogenic capacities exhibited by these cells by targeting SPRED-1, a similar mechanism described for miR-126 delivered by EC-MSCs [24,45]. Hypoxia can also be chemically mimicked by treating MSCs with dimethyloxaloylglycine (DMOG), an inducer of the expression of HIF-1α, an essential transcription factor driving the adaptive cell response to hypoxia. In this line, DMOG-treated MSCs secreted proangiogenic EVs, which promoted the neovascularization and enhanced bone regeneration in animal models of critical-size defects [90].

#### 3.1.2. Engineering of Parent Cells

**Figure 3:**
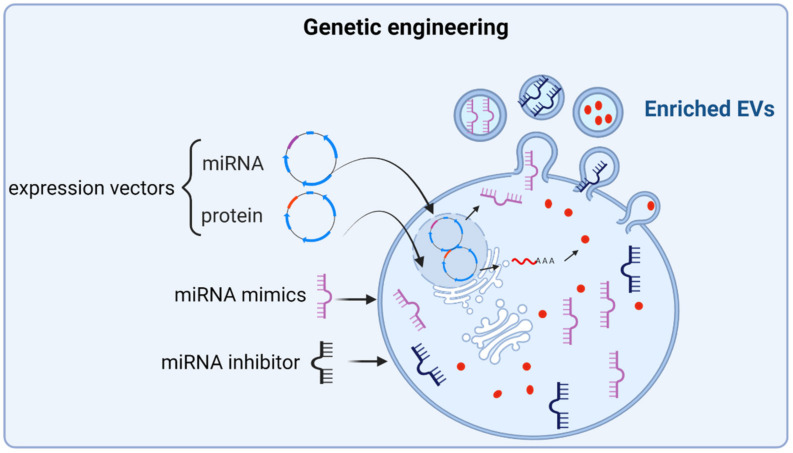
Genetic engineering as an approach to enrich EVs with osteoanabolic factors. The induced expression of known osteoanabolic miRNAs, proteins, or inhibitors of anti-osteogenic miRNAs in MSCs, by using expression vectors or direct transfection approaches of these molecules, yields EVs enriched in these molecules. The figure was created with BioRender.com (accessed on 1 December 2021).

MSCs can be genetically modified to increase the expression of certain pro-osteogenic molecules with the assumption that, this way, their secreted EVs would also be enriched in those induced molecules, enhancing their osteoanabolic properties (Figure 3). In fact, this hypothesis has been validated by overexpressing pro-osteogenic miRNAs in MSCs, such as miR-375 and miR-101. The authors demonstrated that EVs could be enriched in these miRNAs when overexpressed in parent MSCs, without affecting distinctive features of EVs such as morphology, size, and the expression of surface proteins CD9 and CD63, which are used as EV markers. Moreover, these EVs improved the osteogenic differentiation of MSCs and enhanced bone regeneration in animal models of bone defects [57,91].

The overexpression of pro-osteogenic proteins in parent MSCs has also been considered as a strategy to enhance the osteoanabolic potential of EVs. Thus, MSCs overexpressing Osteoactivin (OA), a pro-osteogenic transmembrane glycoprotein, has been shown to secrete OA-enriched EVs, which in addition to enhancing osteogenic differentiation in MSCs, ameliorated OP phenotype in the OVX murine model [92]. Along this line, the induction of the expression of the transcription factor HIF-1α in MSCs led to the production of EVs that not only stimulated osteogenesis of MSCs and angiogenesis of ECs in vitro, but also enhanced bone regeneration in rabbit models of GIONFH by increasing the vascularization of the injured bone tissue [93].

Intriguingly, not always is observed an enrichment of a protein in the EVs cargo after overexpressing that protein in the parent cells. This observation has been recently reported for the pro-osteogenic protein BMP2 [79]. Thus, Huang and colleagues found that, unexpectedly, the EVs secreted by MSCs overexpressing BMP2, although similar to those secreted by unmodified MSCs in terms of general features, such as size and concentration, did not contain BMP2. However, these EVs derived from BMP2 overexpressing MSCs exhibited increased bone regeneration abilities in rat calvarial defect models. In vitro experiments showed that the osteoanabolic capacity of these EVs was due to an enrichment in miRNAs targeting the expression of SMURF1 and SMAD7 in recipient MSCs, known inhibitors of the BMP2 pathway.

EVs express specific proteins on their surface, such as tetraspanins, namely C9 and CD81, that mediate the EVs’ uptake and downstream intracellular signaling on recipient cells by ligand-receptor interactions [94]. These surface proteins can mediate the direct binding of EVs to specific receptors on target cells, triggering cell signaling without delivering their cargo or alternatively, can take part on receptor-mediated endocytosis of EVs, and transfer their cargo to the acceptor cells [19]. These methods of receptor-mediated targeting and uptaking of EVs offer the possibility of engineering EVs to express certain surface proteins known to target tissue-specific cells. One such strategy that has been addressed by genetically engineering NIH-3T3 cells (mouse embryonic fibroblasts) to overexpress CXCR4, a chemokine receptor involved in the mobilization of MSCs towards bone fracture sites [95]. Hu and collaborators revealed that CXCR4 was expressed on the surface of secreted EVs and these CXCR4+ EVs gathered in the bone marrow of long bones when performing in vivo tracing tests in mice [96]. Accordingly, a strong candidate protein to be tested with this approach could be fibronectin (FN), an ECM and cell surface glycoprotein. Interestingly, a very recent study indicated that patients who suffered traumatic brain injury exhibited accelerated bone healing, which in turn was mediated by circulating EVs of brain origin. Strikingly, these EVs exhibited a trend to accumulate in bone, in addition to an enrichment in the expression of FN on their surface, suggesting that FN could be driving the targeting of EVs to bone tissue and therefore accelerating bone repair [97]. Supporting this assumption, previous works found FN expression on the surface of EVs secreted by other cell types, such as ECs, and that this FN mediated the uptake of EVs by binding to heparin sulfate proteoglycans of target cells [98].

### 3.2. Directing EVs to Target Bone Tissue

**Figure 4:**
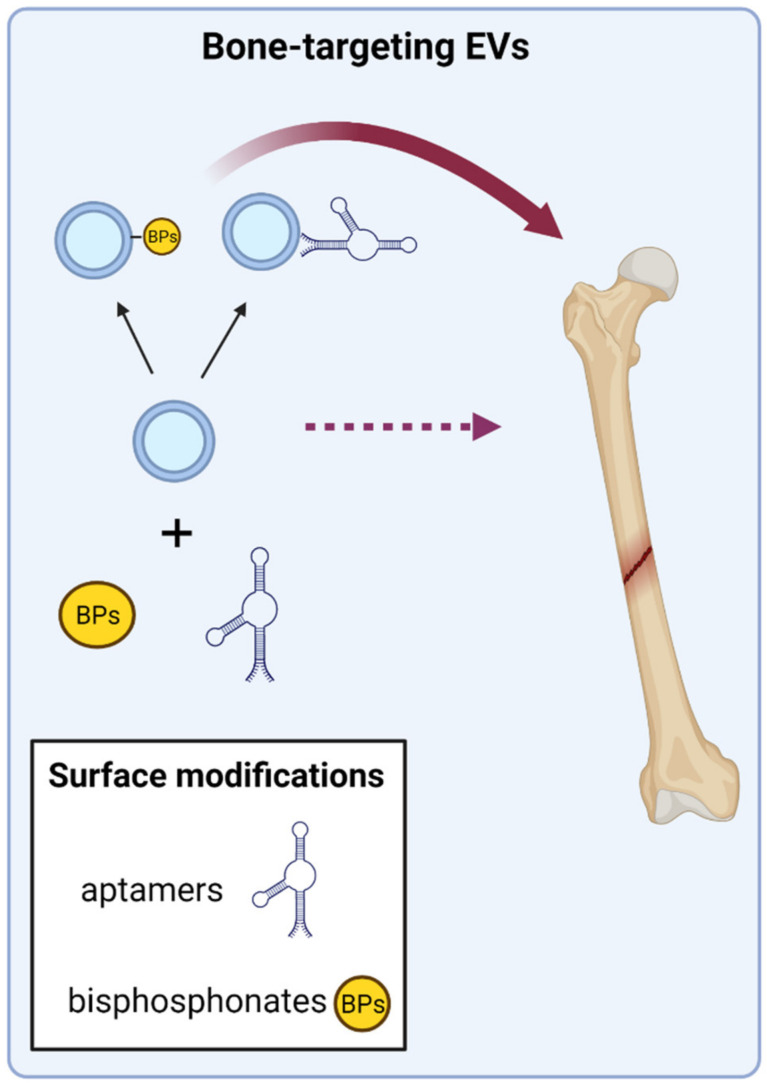
Functionalization of EVs’ surface to improve bone targeting. The surface modification of EVs with molecules showing affinity for bone cells has been described. This is the case of specific aptamers, DNA/RNA molecules with affinity for a desired target, and in this case MSCs and osteoblasts. Anti-resorptive drugs, such as bisphosphonates (BPs), which show high affinity for the mineralized bone matrix, have also been covalently bound to the surface of EVs. Both approaches have demonstrated increased bone targeting of functionalized EVs. The figure was created with BioRender.com (accessed on 1 December 2021).

EV therapy to treat skeletal conditions can be delivered by local or intravenous administration, and each one has their specifications and advantages/disadvantages. The local administration, most suitable for concrete bone fractures or defects, ensures the bone targeting of EVs, but requires the concomitant use of scaffolds, such hydrogels, in order to maintain the EVs in the site of injury. On the contrary, when considering global skeletal conditions, such as OP or OI, the intravenous administration of EVs is the considered option; nevertheless a major drawback of this administration route is the low bone tropism that EVs show. Some innovative strategies are currently under intensive research in order to achieve and enhance the bone targeting of EVs; especially promising are those that directly modify the EVs’ surface with specific bone-targeting molecules (Figure 4).

#### 3.2.1. Aptamer-Guided EVs

Aptamers, short single-stranded DNA/RNA oligonucleotides that function as “synthetic antibodies” have been recently tested to target EVs to bone. Aptamers are synthesized and selected through an in vitro process first developed in 1990 called SELEX (systematic evolution of ligands by exponential enrichment) from a large random sequence library. They are capable of folding, forming unique tertiary structures that show high binding affinity and specificity to targets, which can be molecules, cells, and more recently described EVs [99,100]. Interestingly, several works describe specific aptamers targeting MSCs and osteoblasts, thus opening new avenues to develop specific delivery systems using aptamers with bone anabolic purposes [101,102]. Moreover, two recent works addressed the combination of bone cell specific aptamers with the surface proteins of EVs with encouraging results. Thus, these functionalized aptamer-EVs were able to successfully deliver their cargo to bone target cells in vitro [103,104]. Furthermore, after intravenous administration in mice, the authors showed an improved in vivo bone-targeting and functionality of these aptamer-EVs, which promoted bone regeneration in OVX mice and in mice models of bone fracture [103,104].

#### 3.2.2. Coupling of EVs to Bone-Targeting Drugs

Other approaches are focused on taking advantage of the affinity of certain drugs to bind the mineral phase of bone tissue, such as the anti-resorptive drugs used to treat OP. This way, the surface functionalization of EVs with these drugs to specifically target bone tissue is a strategy that has been recently addressed [105]. Thus, a recent work used copper-free click chemistry to combine the surface of mouse MSCs-EVs with alendronate, a bisphosphonate that binds hydroxyapatite crystals. Upon administration into OVX rats, these Ale-EVs showed no toxicity and increased affinity for bone tissue when compared to control EVs. In addition, the functionality of Ale-EVs was also demonstrated since OVX rats exhibited improvements in bone tissue microstructure after Ale-EVs treatment [106].

## 4. Conclusions

The increasing knowledge about EV biology has strengthened the idea that EVs hold great potential to be applied with therapeutic purposes, mainly due to their ability to transfer diverse bioactive molecules modifying the fate of recipient cells. Thus, EVs may offer a promising “cell free” advanced therapy as next-generation biocompatible vehicles delivering therapeutic factors.

However, before EVs move forward to the clinic, it is mandatory to address several requirements that challenge their claimed therapeutic abilities, including standardization and scalability production, their full molecular characterization, and bioengineering improvements that increase their therapeutic potency. Moreover, as mentioned in this review, the inherent biology of the target tissue plays a key role in the success of EV-based therapies. When intended for bone regeneration purposes, EV therapeutics have to overcome two main limitations, both matters of intense research: the osteoanabolic properties of EVs, which should be enhanced in order to achieve robust, in vivo bone regeneration and the limited tropism for bone tissue that the osteoanabolic EVs show upon administration. Therefore, to achieve bone regeneration, the ideal EVs should combine features aiming to counteract these two limitations.

EVs isolated from a wide range of bone-relevant cells have demonstrated osteoanabolic potential. However, the fact that the majority of studies only rely on EVs isolated from a single cell type hinders the comparison of their osteoanabolic capacity. Therefore, the systematic analysis of different EVs isolated from different cell types abdcomparing their osteogenic capacity should be a prerequisite to identify those EVs, or their combination, with the maximum osteoanabolic potential. This knowledge will come along with the understanding of the mode of action of EVs, and to achieve it, essential requirements should be considered, such as deciphering the molecular players driving the downstream signaling of EVs in target cells. Accordingly, comprehensive multi-omic technologies have enabled a deep characterization of EV cargo, but the identification of those molecular drivers in EVs conducting bone regeneration is just beginning to emerge. So far, the majority of the current research has identified several single molecules, especially miRNAs and some proteins, as drivers of the EVs downstream regulation in the recipient cells. However, considering that EVs carry an array of molecules and that EVs from different cell sources achieve the induction of bone regeneration, it is more likely for a synergistic collaboration of different molecules in target cells to occur, as opposed to a single upstream molecular regulator. In fact, recent evidence point to this observation: Lee and collaborators reported that AT-MSCs-EVs attenuated bone loss in OVX mice by the simultaneous transfer of proteins and miRNAs targeting osteoclasts. Thus, the inhibition of osteoclastogenesis elicited by AT-MSCs-EVs and the subsequent restoration of bone mass in OVX mice was mediated by the transfer of osteoprotegerin (OPG), a decoy receptor for RANK ligands that inhibits osteoclasts differentiation, and miR-21-5p and let-7b-5p, which reduced osteoclast differentiation [78]. Liu and coworkers also identified a multi-component pro-osteogenic miRNAs cargo in BM-MSCs-EVs: let-7a-5p, let-7c-5p, miR-328a-5p, and miR-31a-5p. These miRNAs were shown to synergistically mediate the osteoanabolic properties of BM-MSC-EVs by promoting the activation of the canonical BMP signaling pathway [107].

The increasing knowledge about the most suitable EV cell source and the bioengineering approaches under development will address the aforementioned limitations facilitating the development of EV-based therapeutics that will transform the pharmaceutical scene for bone regeneration. Currently (as of December 2021), there is one clinical trial testing EVs, specifically exosomes, as therapeutic drugs applied for a bone disease: a phase I trial evaluating intra-articular injections of a single dose allogenic MSCs-derived exosomes for knee osteoarthritis (ExoOA-1; NCT05060107). We anticipate that, as different approaches demonstrate improvements in the osteoanabolic potential and bone-targeting abilities of EVs, there will be increasing clinical trials evaluating the safety and potential of this advanced therapy for bone regenerative purposes in the not-so-distant future.

## Figures and Tables

**Table 1:** Main cell sources that secrete osteoanabolic EVs and the identified cargo mediating bone regeneration in different mice models of bone disease.

EVs Source	Bioactive Cargo	Disease Model	Target Molecule-Pathway	Target Process	Ref.
*BM-MSCs*	miR-335	Bone fracture	VAPB-WNT/β-CATENIN	↓ Osteoclastogenesis & ↑ Osteogenesis	[35]
	miR-25	Bone fracture	SMURF1-RUNX2	↑ Osteogenesis	[36]
	NID1	Femoral defects	Myosin-10	↑ Angiogenesis	[37]
	mir-29a	Wild type mice	VASH1	↑ Angiogenesis & ↑ Osteogenesis	[38]
*Aged BM-MSCs*	mir-128-3p	Bone fracture	SMAD5	↓ Osteogenesis	[39]
*UC-MSCs*	miR-1263	Disuse OP	MOB1-HIPPO	↓ Apoptosis	[40]
	miR-21	GIONFH	PTEN-PI3K/AKT	↓ Apoptosis	[41]
	miR-365a-5p	GIONFH	SAV1-HIPPO	↑ Osteogenesis	[42]
	miR-3960	Senile OP	unknown	↑ Osteogenesis & ↑ Osteoclastogenesis	[43]
	CLEC11A	OVX-OP, Disuse OP, Senile OP	unknown	↑ Osteogenesis & ↓ Osteoclastogenesis	[44]
*Hypoxia-UC-MSCs*	mir-126	Bone fracture	SPRED-1	↑ Angiogenesis	[45]
*ECs*	miR-126	Distraction osteogenesis	SPRED-1	↑ Osteogenesis & ↑ Angiogenesis	[24]
	miR-155	OVX-OP	Spi1, Mitf, Socs1	↓ Osteoclastogenesis	[46]

## Data Availability

Not applicable.
